# A first case of successful using of ibrutinib in treating paraneoplastic pemphigus related bronchiolitis obliterans concurrent with CLL

**DOI:** 10.3389/fmed.2023.1132535

**Published:** 2023-03-15

**Authors:** Can Chen, Ying Xu, Jingdi Yu, Shenxian Qian, Yaping Xie

**Affiliations:** Department of Hematology, Affiliated Hangzhou First People’s Hospital, Zhejiang Chinese Medical University, Hangzhou, China

**Keywords:** bronchiolitis obliterans, chronic lymphocytic leukemia, ibrutinib, paraneoplastic pemphigus, mechanism

## Abstract

Paraneoplastic pemphigus (PNP) is a rare life-threatening disease which always associated with an underlying neoplasm. Tumor-related PNP most commonly precedes the detection of a hematological malignancy, with some cases seen during disease remission following cytotoxic drug therapy or radiotherapy. The lung is the most frequently-involved site in PNP, second only to the eyes, and involvement is seen in 59.2% to 92.8% of PNP cases. Bronchiolitis obliterans (BO) is the end stage of respiratory involvement and is regarded as life-threatening. The key point in treatment of PNP is to control the associated underlying hematologic neoplasia. High-dose systemic corticosteroids combined with other immunosuppressants are considered the first line of treatment. Other therapies that have shown beneficial effects include plasmapheresis, intravenous immunogloblin (IVIG), and more recently, daclizumab, alemtuzumab, and rituximab. There is no effective treatment for BO with PNP, and suppression of the cellular immune response may be necessary. Patients with PNP-BO associated with lymphoma mostly die within approximately 1 year. Herein, we reported a patient who diagnosed with PNP-BO concurrent with chronic lymphocytic leukemia. He was successful treated with ibrutinib and had achieved the longest survival which suggested that ibrutinib may be the best treatment choice for such patient.

## 1. Introduction

Paraneoplastic pemphigus (PNP) is an autoimmune bullous disease associated with an underlying neoplasm, mainly a lymphoproliferative disease, such as chronic lymphocytic leukemia (CLL) (1). Tumor-related PNP most commonly precedes the detection of hematological malignancy, with some cases occurring during disease remission following cytotoxic drug therapy or radiotherapy (2). This may account for the underlying pathogenesis of PNP, as tumor cells may produce specific humoral and cell-mediated immune responses contributing to the development of PNP manifestations.

Initial studies have suggested that tumors induce autoantibody production against self-antigens, dysregulation of immune function, immune cross-reactivity against epithelial antigens, and elevated IL-6, leading to increased B-cell differentiation, cytotoxic T-cell activation, and antibody production (3). The primary clinical manifestations of PNP are mucocutaneous lesions that are polymorphous, widespread, and frequently severe and resemble pemphigus vulgaris, erythema multiforme, lichen planus, and graft-versus-host disease (1, 4). Bronchiolitis obliterans (BO) is a fatal disease that affects approximately 20% of PNP who also have CLL. There has yet to be a published case that describes an effective treatment for PNP-BO secondary to CLL. In the context of CLL, as in this case, ibrutinib may be the first therapeutic option to save a patient’s life.

## 2. Case presentation

**Figure 1:**
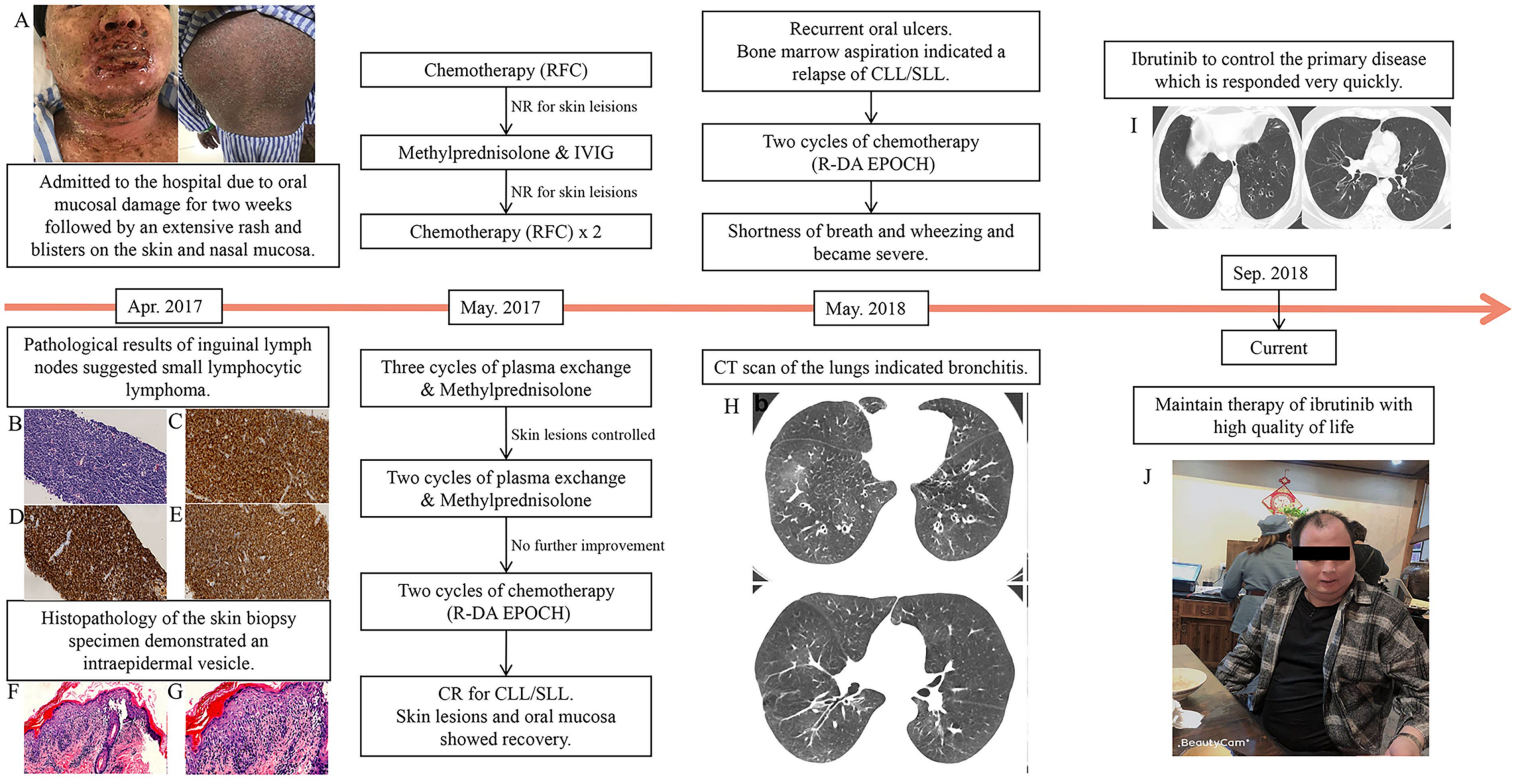
The patient’s clinical evaluation and treatments. **(A)** Pemphigus lesions on the skin and lip were extremely severe before treatment. A lymph node biopsy revealed **(B)** small B-cell lymphoma (magnification, ×400). Positive immunohistochemical results were observed for **(C)** CD5 (magnification, ×400), **(D)** CD20 (magnification, ×400), and **(E)** CD23 (magnification, ×400). Skin biopsy specimens demonstrating intraepidermal vesicle [**(F)** (magnification, ×200)]. During the treatment of the relapse period, the patient developed shortness of breath and wheezing. **(G)** At the onset of bronchiolitis obliterans, high-resolution computed tomography revealed hyperinflation and bronchial wall thickening in bilateral lungs. **(H)** After treatment, the bronchial wall thickening was alleviated. **(I)** Currently, the skin lesions are healing and the patient is in relatively good health **(J)**.

On April 6, 2017, a 36-year-old man was admitted to the hospital for 2 weeks of oral mucosal damage, which was followed by an extensive rash and blisters on the skin and nasal mucosa. His skin was covered in red papules, and there was some skin exfoliation, especially on his chest, back, and limbs. Additionally, there was conjunctival hyperemia and fissuring of the lips with scabs. The oral mucosa was covered by a white exudate, and there was ulceration that bled easily. A slight erosion of the anus was also detected (Figure 1A).

Blood routine was performed with white blood cell counts of 37.6 × 10^9/L (normal range 3.5–9.5 × 10^9/L), hemoglobin level of 148 g/L (normal range 130–175 g/L) and platelets counts of 230 × 10^9/L (normal range 125–350 × 10^9/L). A systematic immunological assessment was performed which were all negative (antinuclear antibodies, antineutrophile cytoplasmic antibodies, anti-mitochondrial antibody, anti-U1-nRNP antibodies, anti-Sm antibodies, anti-SS-A antibodies, anti-SS-B antibodies, anti-Ro-52 antibodies, anti-Scl-70 antibodies, anti-PM-Scl antibodies, anti-Jo-1 antibdies, anti-centromere antibodies, anti-double-stranded DNA antibodies). The tests indicated infection such as C reactive protein, procalcitonin, copies of EBV and CMV, cryptococcus capsular polysaccharide antigen, 1,3- β- D-glucan test and galactomannan test were negative as well. Other tests, such as lactic dehydrogenase(292 U/L, normal range 50–240 U/L), β2-microglobulin (2,416 μg/L, normal range 1,010–1730 μg/L) were slightly high. Subsequently, bone marrow aspiration and biopsy were performed. Bone marrow aspiration revealed a high proportion of lymphocytes and immature lymphocytes accounting for 4.5% of the total cells. Bone marrow biopsy indicated infiltration of lymphocytic leukemia cells. A flow cytometric study of both the bone marrow and peripheral blood revealed an abnormal proportion of lymphocytes, accounting for 69.0 and 66.3% of the nucleated cells, respectively. Flow cytometry study of peripheral blood revealed high proportion of lymphocyte with positive expression of HLA-DR, CD19, CD200, clambda, partial were positive with CD5, CD20(dim), CD22(dim), CD23, and with negative expression of CD10, CD11c, CD25, CD34, CD38, CD103, Bcl-2, FMC-7. Flow cytometry study of bone marrow revealed an abnormal proportion of lymphocyte, of these, CD19+ cells were 61% of nuclear cells with positive expression of HLA-DR, CD19, CD200, clambda, partial were positive with CD5, CD20(dim), CD22(dim), CD23, slambda(dim), and with negative expression of CD10, CD11c, CD25, CD34, CD38, CD103, Bcl-2, and FMC-7. These abnormal cells were considered chronic B lymphocytic leukemia (B-CLL) cells. Fluorescence *in situ* hybridization (FISH) tests for 11q22, and 13q14 deletions were positive, meanwhile deletion of TP53(17p13.1) and rearrangement of BCL-2, CCND1, C-MYC were negative. Other CLL investigations such as the mutation status of IgVH was negative. Positron emission tomography-computed tomography (PET-CT) revealed swelling of multiple lymph nodes, indicating lymphoma.

Based on the results of PET-CT, we chose inguinal lymph nodes for biopsy. The pathological findings suggested that the patient had small lymphocytic lymphoma (SLL; Figures 1B–E). Since this patient presented with severe oral mucosal ulceration and skin lesions, we performed a skin biopsy (Figures 1F,G). Histopathology of the skin biopsy specimen demonstrated an intraepidermal vesicle. Direct immunofluorescence revealed the presence of complement C3, indicating the presence of PNP. Serum antibodies against desmoglein-3, the key target antigen in pemphigus vulgaris and pemphigus foliaceus, were also found to be positive. On May 20, 2017, the patient received chemotherapy for SLL that included rituximab in combination with fludarabine and cyclophosphamide (FCR).

After one cycle of chemotherapy, there was no obvious improvement in the skin lesions. We increased the dosage of methylprednisolone and added intravenous immunoglobulin, but still, there was no improvement. The second and third cycles of FCR chemotherapy were administered on June 12, 2017, with no response from the skin lesions. As a result, we performed five cycles of plasma exchange and began administering methylprednisolone on a daily basis (dosage of 80 mg daily). His skin lesions appeared to be under control after three cycles of plasma exchange. However, no further improvement in the skin lesions was seen in the last two cycles.

Two cycles of standard chemotherapy consisting of rituximab in combination with dose-adjusted etoposide, vindesine epirubicin, cyclophosphamide, and dexamethasone (R-DA EPOCH, Rituximab 700 mg for d0, etoposide 0.075 g daily for d1-4, vindesine 1 mg daily for d1-4, epirubicin 25 mg daily for d1-4, cyclophosphamide 1.3 g daily for d5, and dexamethasone 30 mg daily for d1-5) were administered. We performed a systematic evaluation after two cycles of chemotherapy, which revealed complete remission. Furthermore, the skin lesions and oral mucosa recovered. We recommended that the chemotherapy be continued or that an allogeneic hematopoietic stem cell transplant be performed. However, due to financial concerns, the patient declined treatment and was discharged.

He returned to our hospital in May 2018 with recurrent oral ulcers. A bone marrow aspiration revealed that leukemia had relapsed. As a result, we recommended ibrutinib treatment, but the patient declined. Another two cycles of DA-EPOCH were administrated to the patient. During chemotherapy, he reported shortness of breath and wheezing. Pulmonary function tests revealed extremely severe obstructive as well as restrictive lung disease, with a moderate decrease in the diffusion volume and a slight decrease in diffusion (%VC = 64.4, %FEV1.0 = 23.4, %FEV1.0 improved 3.9 after inhalation of bronchodilator). Blood gas analysis revealed no abnormalities. Shortness of breath became severe 1 week later, and an arterial blood gas analysis revealed type I respiratory failure. A CT scan of the lungs revealed bronchitis (Figure 1H). Severe BO was considered after a multidisciplinary consultation. Supportive care was maintained, and lung transplantation was once considered. His situation improved very quickly after 2 weeks of using ibrutinib (420 mg oral per day), which was unexpected and surprising. After 1 month, the patient recovered and was discharged with ibrutinib continuous therapy. He recovered well after that with ibrutinib maintenance therapy (Figure 1I). He has been disease-free and has had a relatively high quality of life for 30 months (Figure 1J).

## 3. Discussion

The lung is the second most commonly involved site in PNP, after the eyes, and involvement is seen in 59.2 to 92.8% of PNP cases. BO is the terminal stage of respiratory involvement and is considered fatal because it can cause acute irreversible pulmonary failure. Due to the paucity of data on PNP-related BO, the precise pathologic mechanism underlying BO development has not been elucidated; however, several hypotheses have been proposed, with studies concluding that it is likely the result of autoantibodies against plakin and desmoglein proteins in the skin and respiratory epithelium. Epiplakin, one of the plakin proteins, has been linked to BO development and has been linked to increased mortality. Furthermore, anti-epiplakin autoantibodies have been found in the vast majority of PNP sera (3). These antibodies were produced by B cell clones or reactive B cells (4), and they were capable of recognizing antigens expressed in stratified squamous epithelia and transitional columnar epithelia. Immunohistochemical analysis revealed that infiltrating CD8+ T lymphocytes invade the bronchiolar walls, suggesting that cell-mediated cytotoxic mechanisms, particularly CD8+ T lymphocytes, may contribute to the progression of BO (5). Immune mechanisms that cause BO are thought to trigger inflammatory changes such as the nitric oxide (NO) pathway and an increase in inflammatory cytokines (6). Profibrotic mediators, such as transforming growth factor-b, vascular endothelial growth factor, fibroblast growth factor, and platelet-derived growth factor, are elevated during and may play a significant role in BO as well (6–10).

The diagnostic algorithm for PNP has been updated since Anhalt first described it in 1990 (5). Several case reports have demonstrated that PNP is associated with a poor prognosis and a high mortality rate of 90% at 2 years, usually due to secondary systemic complications, especially sepsis, gastrointestinal bleeding, and BO (6). The key point in PNP treatment is to control the underlying hematologic neoplasia. The first line of treatment is high-dose systemic corticosteroids combined with other immunosuppressants. Other therapies that have shown beneficial effects include plasmapheresis, immunogloblin (IVIG), and more recently, daclizumab, alemtuzumab, and rituximab (7). Ibrutinib, a BTK inhibitor that reduces B-cell proliferation and the production of proinflammatory cytokines, has been shown to be an effective treatment option in patients with B-CLL/SLL-associated PNP (8). Nonetheless, a recent study found that patients treated with ibrutinib did not have sustained responses, leading to the hypothesis that rituximab combined with ibrutinib may produce better results than single-agent ibrutinib in patients with B-CLL/SLL-associated PNP (9).

**Figure 2:**
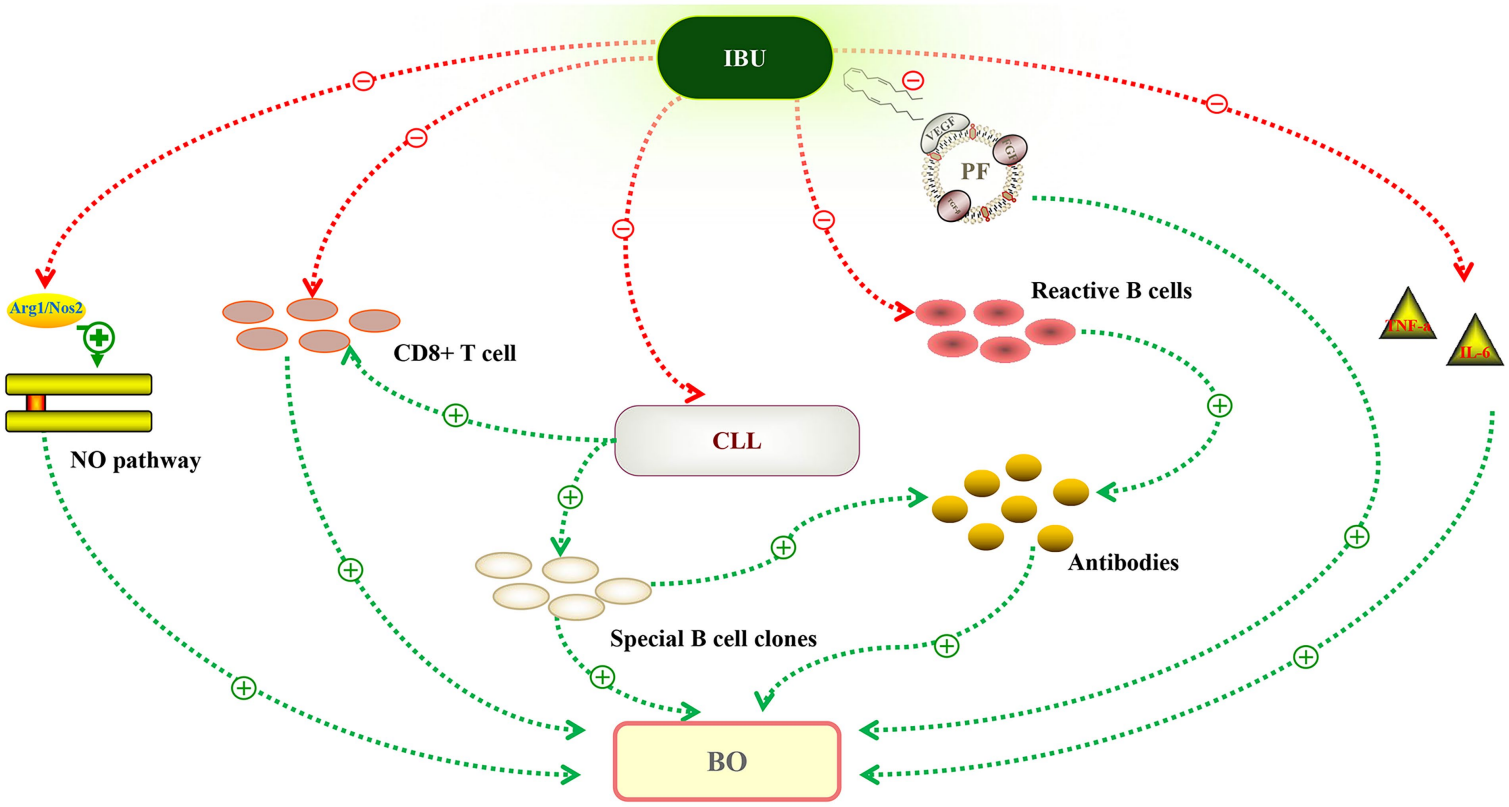
Potential mechanism of ibrutinib in the treatment of bronchiolitis obliterans (BO). Autoantibodies against plakin and desmoglein proteins could recognize the antigens expressed in stratified squamous epithelia and transitional columnar epithelia. Ibrutinib reduced the production of antibodies by inhibiting special B cell clones or reactive B cells. Chronic lymphocytic leukemia (CLL) cells may impair T-cell effector function, leading to a proliferation of infiltrating CD8+ T lymphocytes invading the bronchiolar walls. Ibrutinib targets CLL, and after treatment, CD8+ T cells decreased significantly. The nitric oxide (NO) pathway plays a significant role in BO, and ibrutinib treatment decreased NO production by downregulating Nos2 expression. Ibrutinib may also inhibit inflammatory changes, including the inflammatory cytokines (IL-6, TNF-a) and vascular endothelial growth factor (VEGF). Green line, promote. Red line, inhibit.

Ibrutinib is an irreversible inhibitor of Bruton’s tyrosine kinase (BTK) that targets B cells and induces long-term remissions in B cell malignancies, especially CLL/SLL. CLL cells may cause chronic activation of T cells and impairment of T-cell effector function. Ibrutinib treatment may alleviate the disease burden and immune dysfunction associated with CLL. A dense lymphohistiocytic infiltrate, particularly CD8+ T cells, and linear deposition of IgG and complement along the lamina propria, as well as acantholysis of the bronchial epithelium, have also been implicated in BO (11). A previous study revealed that ibrutinib significantly reduced regulatory T cells and CD8+ T cells (12). In murine models of BO, ibrutinib treatment could reduce germinal center reactions and tissue immunoglobulin deposition (13). Ibrutinib can also inhibit the production of inflammatory cytokines such as IL-6, IL-10, and TNF-α and form a covalent bond with EGFR C797 (14, 15). Ibrutinib treatment decreased nitric oxide synthase-2 (Nos2) expression and NO production, potentially altering the NO pathway. All of this could explain the potential mechanism (Figure 2) of this patient’s unexpected response to ibrutinib treatment.

Patients with PNP-related BO associated with lymphoma mostly die within a year. Due to severe infections, poor performance status, respiratory failure, and underlying lymphoma, they do not receive enough immunochemotherapy. There is no standard treatment for such patients; however, intensive immunochemotherapy may be beneficial in improving the prognosis, though it cannot prevent lethal respiratory failure. This patient’s treatment was a huge success. After intensive chemotherapy, the longest survival time for such patients was 27 months. The response to ibrutinib in this patient was unexpected. As a result, we recommended that he get a lung transplant. His disease was under control during the preparation period, and he recovered quickly. As a result, he can be said to have had the best outcome of any similar patient.

To summarize, the mechanism of BO in PNP is unknown, and our patient is the longest survivor with a promising prognosis. Due to our limited understanding of this unusual disease, we are unable to explain the mechanism. Ibrutinib may be a useful treatment option for patients with PNP-related BO associated with lymphoproliferative disorders, particularly CLL/SLL.

## Data availability statement

The original contributions presented in the study are included in the article/supplementary material, further inquiries can be directed to the corresponding authors.

## Ethics statement

Written informed consent was obtained from the individual(s) for the publication of any potentially identifiable images or data included in this article.

## Author contributions

YaX and SQ were responsible for the study design. CC, JY, and YiX were responsible for formal analysis and investigation and data curation. CC and JY contributed significantly to the writing of the manuscript. YaX, SQ, and YiX were major contributors in coordinating patient care. CC, JY, and SQ provided discussion, critical feedback, and manuscript editing. All authors contributed to the article and approved the submitted version.

## Funding

This work was supported by Hangzhou Science and Technology Plan (grant no: 202004A15) and Hangzhou Health Science and Technology Project (grant no: Z20210039).

## Conflict of interest

The authors declare that the research was conducted in the absence of any commercial or financial relationships that could be construed as a potential conflict of interest.

## Publisher’s note

All claims expressed in this article are solely those of the authors and do not necessarily represent those of their affiliated organizations, or those of the publisher, the editors and the reviewers. Any product that may be evaluated in this article, or claim that may be made by its manufacturer, is not guaranteed or endorsed by the publisher.
